# Multidisciplinary early activity team collaboration practice in ICUs: A qualitative study

**DOI:** 10.1371/journal.pone.0330516

**Published:** 2025-08-26

**Authors:** Xueqin Wang, Jie Mi, Chuanlin Zhang, Ruiying Gan, Xinyi Luo, Qinghua Zhao

**Affiliations:** 1 Department of Critical Care Medicine, The First Affiliated Hospital of Chongqing Medical University, Chongqing, PR China; 2 Nursing Department, The First Affiliated Hospital of Chongqing Medical University, Chongqing, PR China; Neighborhood Physical Therapy, UNITED STATES OF AMERICA

## Abstract

**Purpose:**

To understand the collaborative practice experience of ICU patients’ multidisciplinary early activity team members to provide a reference for formulating a scientific multidisciplinary team collaboration model.

**Methods:**

Based on the phenomenological research methodology of qualitative research, in-depth interviews were conducted with 22 multidisciplinary early mobilization teams of ICU patients in nine general tertiary hospitals in China, using a purposive sampling method, Themes were then distilled.

**Results:**

Three themes and six subthemes emerged from the analysis. (1) Inadequate collaboration concepts: ① manager's philosophy, ② multidisciplinary membership concept; (2) Poor team building: ① poor team structure, ② member training needs to be strengthened; (3) Imperfect team management mechanism: ① no continuous improvement measures, ② unclear incentives.

**Conclusion:**

Good teamwork, which promotes early mobilization of ICU patients, is a scientifically feasible model of collaboration that should be constructed to guide the team in their work.

## Introduction

Insufficient physical activity in ICU patients leads to muscle weakness, delirium, and prolonged mechanical ventilation, and these complications persist years after ICU discharge [1]. Multidisciplinary early activities [2–3]within 72 hours of ICU admission [4–5], the early mobility team, composed of doctors, ICU nurses, and rehabilitation therapists, worked together to formulate a systematic and personalized early mobility plan for patients and implement it. At the same time, the plan was discussed and adjusted by multidisciplinary teams. Studies have confirmed that the effect of multidisciplinary early mobilization is superior to that of routine early mobilization groups [6], and the success of multidisciplinary early mobilization depends on effective teamwork [7]. However, affected by many factors, early ambulation teams often face the problem of poor collaboration, which directly affects the development of early ambulation of patients [8]. Effective implementation of early ambulation in ICU patients should increase the dose of rehabilitation therapy and consider the necessary teamwork and cultural intervention [9].

According to Mukpradab [10], structured teams for early activity in ICU patients most often included nurses (97.3%), physiotherapists (86.5%), doctors (75.7%), respiratory therapists (48.6%), occupational therapists (35.1%), pharmacists (21.6%), technicians (8.3%), nursing assistants (8.3%), and senior practice staff (8.3%). However, staffing structures do not facilitate early activity work, and collaborative staffing programs may be more helpful. Team strategies facilitate the implementation of early mobilization, such as defining team composition and disciplinary roles [11], providing education, reminders, audits, feedback strategies [12], adequate calming assessments and delirium management [13], etc. In practice, the implementation of multidisciplinary early activities was also challenged by the absence of clear team responsibilities and leaders, low levels of staff knowledge and practical skills, negative staff attitudes, high staff workloads, and poor communication [14–16]. Specifically, the optimal team composition, disciplinary roles, and responsibilities included in this approach are unclear and require extensive experimental research to explore and validate [17].

In the previous study [18], our research team used a multi-center cross-sectional survey to understand the implementation situation of multidisciplinary early activities in ICUs across China and analyze the influencing factors. It is found that most of the multidisciplinary teams cooperation in ICU early mobility in China faces the problems of lack of standardized programs, unclear division of responsibilities, and unclear multi-professional and multidisciplinary team. However, the specific processes undertaken remain unclear, including the perspectives of multidisciplinary team members and the challenges encountered during implementation. Quantitative research has limitations in understanding the deep motivations of healthcare workers, decision-making processes, and the complex ethical and sociocultural barriers that may be encountered in team collaboration. Therefore, it is necessary to conduct qualitative research to explore the experiences, perspectives, and challenges of multidisciplinary team members in early mobility practice through in-depth interviews, so as to more fully understand the complex factors affecting early mobility implementation and provide more targeted recommendations for future interventions.

## Methods

### Design

Considering that this study was designed to gain insight into the experiences and perceptions of ICU multidisciplinary early mobility team members rather than to make statistical inferences about the overall situation. By purposefully selecting ICU team members from different cities, different hospitals, and different specialties, richer and more comprehensive information can be collected so as to understand the current situation and challenges of ICU early activities more deeply. Therefore, this study selected general tertiary hospitals in 9 cities in China (including Beijing, Chongqing, Chengdu, Sichuan, Changsha, Hunan, Wuhan, Hubei, Nanjing, Jiangsu, Shijiazhuang, Hebei, Lanzhou, Gansu, and Nanning, Guangxi) that had conducted early activities for ICU patients, using a purposive sampling method.

The study was approved by the ethics committee of the First Affiliated Hospital of Chongqing Medical University (Approval number: 2022–215). Verbal and written informed consent were obtained, and all participants received information regarding their right to withdraw participation without giving a reason.

### Participants and settings

From May 11th to December 3rd, 2023, ICU multidisciplinary early activity team members, including physicians, nurses, and rehabilitation therapists, were recruited. Inclusion criteria: members of a multidisciplinary early mobility team for ICU patients. The sample size was determined using data saturation as the criterion; that is, the interview was stopped when new interview subjects could not provide new information.

### Data collection

Considering the wide scope of this study and the busy work of the research subjects, online interviews can break through the geographical limitations, save time and cost, and improve the research efficiency. Therefore, this study conducts online interviews through the WeChat platform or telephone contact, and the specific methods are voluntarily selected by the respondents, and a good relationship is established with the respondents before the interview. First, the researcher obtained the contact information of potential interview subjects through the hospital contact person and introduced the purpose and significance of the study to them by WeChat platform or telephone to obtain their preliminary consent. Secondly, before the formal interview, the researcher will have a brief online communication with the interviewees to get familiar with each other and establish a trust relationship, and at the same time, make an appointment for the formal interview. During the communication, the researcher will patiently answer the questions raised by the interview subjects and once again emphasize the confidentiality of the research.

The online interview used a one-to-one, half-structured interview to explore the perception, implementation, and challenges of early mobility among multidisciplinary teams in ICUs. Compared with the structured interview, the semi-structured interview allows researchers to flexibly adjust the questions according to the answers of the interviewees on the basis of the preset interview outline so as to explore the information more deeply. The first author used online interviews (telephone, WeChat voice), fully communicated with the interviewees before the interview, informed them of the purpose, content, and method, and promised to follow the principle of confidentiality. After obtaining consent to the recording, the two sides will interview in a quiet and independent environment, and the researchers will take notes and record. Each interview lasts 30–45 minutes.

In order to ensure the quality of the interview, the researcher will agree on the interview time with the interviewees in advance and remind them to choose a quiet and undisturbed environment. During the interview, the researcher will actively ask the interviewees if they are disturbed and pause or adjust the interview according to the situation. At the same time, researchers will prepare alternate plans, such as changing the interview platform or changing to telephone interviews, in case of emergencies.

### Data analysis

After the interview, the audio recordings or transcripts were sorted into drafts and returned to the interviewees for confirmation within 24 hours to ensure accuracy. The interview confirmation drafts of each respondent were ordered. The collected data were summarized and sorted out, and the Colaizzi 7-step analysis method was used in the process of interview data analysis [19]: (1) Read all records carefully; (2) Choose important statements; (3) encode recurring ideas; (4) compile the coded opinions; (5) write a detailed description without omission; (6) identify similar ideas; (7) If there are any questions, return to the respondents for verification.

Data were collected using open-ended questions to ensure credibility. Two researchers read the transcripts independently during the analysis and identified themes and categories. Finally, the researchers compared and contrasted themes and categories. If there was disagreement, debate and discussion were allowed until a consensus was reached.

## Results

22 medical and technical personnel participated in the interview, including nine doctors, ten nurses, and three rehabilitation therapists(N1-N22). The mean age was 37.41(SD = 6.50), and the mean work experience was 13.18(SD = 7.27). (Table 1)

**Table 1:** General Information Of Interviewees (N = 22).

Projects	Classification	Number	Percentage(%)
Gender	Women	13	59.1
	Man	9	40.9
Education	Undergraduate	6	27.3
	Master	12	54.5
	Doctor	4	18.2
Professional Title	Junior	2	9.1
	Intermediate	13	59.1
	Senior	7	31.8

Three themes and six subthemes emerged from the analysis (Table 2). The themes and subthemes are presented below with illustrative quotes.

**Table 2:** Themes And Subthemes.

Themes	Subthemes
Inadequate Collaboration Concept	Manager’s Philosophy
	Multidisciplinary Membership Concept
Poor Team Building	Poor team structure
	Member Training Needs To Be Strengthened
Imperfect Team Management Mechanisms	No Continuous Improvement Measures
	Unclear Incentives

### Theme 1: Lack of teamwork concept

The respondents in this study believed that the cultural atmosphere of the ICU and the collaborative concept of team members were significant factors affecting the rate of early activities in ICU patients, which were also problems that needed to be solved urgently.

“I think this is teamwork, we must work together, but at present, it is far from enough. At least some people have not formed this (collaboration) consciousness.” (N1)

“To carry out this thing (early activity), first of all, the concept of the department director, the head nurse, and the doctor in charge must be updated. It is difficult to achieve this thing without team awareness.” (N2)

“You cannot do it without others’ cooperation. It is a team thing. If someone does not pay attention to it, it cannot be done.” (N5)

“It is not desirable to stay in the stage of doing everything individually. Managers need to think about bringing the team together, which is the key to whether (early activities) can be carried out continuously.” (N4)

### Theme 2: Imperfect team building

#### Subtheme 1: Poor team structure.

Clarifying the responsibilities of the members of the early activities team and giving full play to the advantages of multidisciplinary expertise are essential guarantees for the safety and efficiency of multidisciplinary early activities. In this study, many members reported that their early mobile teams did not have a clear division of labor and clear professional boundaries, and the lack of organizational coordinators made the problem of poor communication among members more common.

“There should still be a core member of the team who initiates this early activity so that it can move forward. The ICU doctor or nurse plays this role. Sometimes, the doctor initiates it, sometimes, the nurse reviews and consulates with the doctor to initiate it, but we do it less normatively and more casually.” (N18)

“I feel like I have to do it myself, no one organizes it, and there is a lack of communication. Disciplines must communicate with each other. Otherwise, how can we get together to do something? It is communication that affects (early activities implementation).” (N20)

“The team should have a leader or organizing personnel, through his influence, let everyone realize that we should do this thing (early activity), all do this thing (early activity), there is a lack of communication between us (medical staff) and rehabilitation therapists, so our activities are not planned and systematic, which is not well done.” (N17)

“Multidisciplinary teams exist, but it seems unclear who should do what. In the patient's activity process, the starting point, the process, the goal, and the endpoint are crucial, and everyone (team members) should know what to do.” (N10)

“When we do early activities, the medical staff will watch at the bedside without saying who is responsible for which part. For example, if the patient's heart rate is relatively fast or is uncomfortable or irritated, we will put him back in the empty bed in time.” (N7)

“In theory, doctors have to assess when patients can do (early mobilization), and then assess the risk of doing (early mobilization), nurses see if the condition is stable, therapists guide them what to do, and everyone is safe together. In practice, it seems less clear.” (N8)

#### Subtheme 2: Member training needs to be strengthened.

Implementing multidisciplinary early activities for ICU patients has high requirements for the knowledge reserve of team members. Therefore, relevant knowledge training and updating are essential. The respondents of this study believe that the training of early mobility members needs to be strengthened.

“Team building, that is, the continuous growth and progress of the team, the need to constantly update the knowledge and practice level, the ability of the team members to meet the standard, and the tacit understanding of the cooperation, otherwise it may affect the rehabilitation effect, which still needs to be strengthened.” (N20)

“I think it is vital to strengthen training, which impacts the efficiency and quality of activities. It requires team members to continue to learn, which is quite a high requirement.” (N17)

“Training is essential. I think we can train a small number of people first and then spread out. Without systematic training, it is not feasible to only let me do it without teaching me.” (N22)

“Instead of everyone not receiving systematic and complete training, I think we can set up a full-time (early activity) team and train them. In this way, the training system and the whole implementation process are relatively standardized so that we can cooperate better and solve part of the problem of insufficient staffing.” (N15)

### Theme 3: Imperfect team management mechanism

#### Subtheme 1: No continuous improvement measures.

Continuous quality improvement is a valuable strategy for promoting the implementation of early mobility in ICU patients [20]. In this study, most respondents believed that continuous quality improvement measures should be developed to ensure the continuity of early activities, but the results were unsatisfactory.

“The most important thing we should reflect on is the lack of reflection. We just concentrate on doing (early activities), and no one seems to care whether it is good or not, how effective it is. We should make a special plan for the patient before the early activities, and then we (team members) follow the plan to implement it and discuss and modify problems in time, using tools such as PDCA to improve it.” (N20)

“If we can make a continuous improvement for some problems existing in the actual implementation process so that our whole process can be carried out continuously and effectively, that would be the best and give us (team members) confidence.” (N21)

“Now, if there are adverse events, people are afraid to continue to do. The improvement and development of a team should not be limited to one or two adverse events, but after the occurrence of adverse events through discussion and summary, similar to brainstorming, to continue to improve. It is important to establish a virtuous circle.” (N17)

“This kind of multidisciplinary team cooperation, if doctors, nurses, and rehabilitation therapists can adjust the clinical treatment plan and rehabilitation activity plan for patients every week or every two weeks, the benefits and effects for patients will be better. I do not understand why it is not organized.” (N16)

#### Subtheme 2: Unclear incentive mechanism.

Some respondents believed that to promote the development of early activities. Appropriate incentive mechanisms could be set up to improve team members’ enthusiasm.

“Department directors and head nurses should create an atmosphere for carrying out early activities and even provide support, such as time guarantee and reward support, which is better to carry out.” (N18)

“If the leaders pay enough attention to it, they should regard this (early activity) as a routine. If several people do it specifically and give corresponding rewards, why don't you do it?” (N22)

“I think it is necessary to introduce a reward and punishment measure. For example, if I do (early activities), it will certainly increase the risk, but if I do not, I will not get any punishment. Whether it is to give some appropriate and reasonable rewards in income, academic, or other aspects, it will better promote (early activities).” (N15)

“On the one hand, I think this (early activity) should be linked to the performance of our doctors and nurses because the workload is too heavy, and every day patients go to the field (activity) needs many people to help, and the enthusiasm should be encouraged.” (N19)

## Discussion

At present, the early activities of multidisciplinary collaboration in China are still in the exploratory stage, and there are still many obstacles in the promotion and practice of early activities, including the relative shortage of human resources, lack of team cooperation experience, and other problems [8]. If we blindly build an early activities team without knowing how to collaborate, it will increase team members’ workload and hinder early activities implementation [21]. Therefore, early activities can be genuinely promoted only by clarifying the actual feelings of team members in the early activity collaboration, strengthening team building from multiple dimensions, and constructing and improving the collaboration plan.

(1) **Improve the teamwork concept of team members**

The safety of early mobilization has been demonstrated in different stages of the disease in ICU patients [22–24]. Due to the complexity of the disease and the fact that early mobilization involves interdisciplinary fields such as critical care medicine, nursing, and rehabilitation medicine, there are many problems in the intervention process. Multidisciplinary team cooperation can enable patients to obtain comprehensive, timely, and effective treatment, care, and rehabilitation and ensure the implementation of early mobilization [25]. The study found that without multidisciplinary collaboration, nurses would be able to provide in-bed or bedside activities to only 21% of ICU patients [26]. Therefore, from the individual level, medical staff at all levels should realize that multidisciplinary collaboration to carry out early activities is necessary to promote the rehabilitation of ICU patients.

From the management level, an important method to improve the concept of early activities of medical staff is to carry out ICU culture construction. Many studies have shown that culture and concept are important factors affecting the development of early activities in ICU patients. However, the construction of culture and concept is often difficult to implement and evaluate the effect [26]. At present, there are many scientific models for the cultural construction of medical personnel [27] and assessment tools for team collaboration [28], and the effectiveness of enhancing cultural awareness, knowledge, and skills of medical staff and promoting positive behavior change has been confirmed [29]. Only by improving internal ideas, carrying out cultural construction from a scientific perspective, and changing from “passive” to “active” can we truly benefit the development of early activities.

(2) **Strengthen the team building of early activities**

1) **Poor team structure**

A clear team structure and a clear division of responsibilities are prerequisites for good teamwork. Doherty [30] found that the unclear division of labor and poor communication among multidisciplinary team members were essential factors that made it challenging to implement the early activities of patients. The responsibilities of multidisciplinary team members in patients’ early activities included pre-activity assessment, activity preparation, activity process implementation and monitoring, team members’ discussion of activity obstacles, activity plan and goal formulation, and activity level and adverse events recording.

Nurses carried out the whole early ambulation assessment, planning, and implementation process [31,32]. Doctors assisted with the assessment process, and rehabilitation therapists were responsible for nurse training and guidance on key points of activities. For the multidisciplinary early mobility team, the division of labor was as follows: the ICU director was responsible for supervising the study implementation; The ICU head nurse was responsible for contacting team members and coordinating teamwork. ICU doctors were responsible for patient evaluation and rehabilitation doctor's advice. The respiratory therapists were responsible for artificial airway management, mechanical ventilation parameter adjustment, and weaning. Rehabilitation therapists and early activity full-time nurses were responsible for evaluating the rehabilitation effect on patients and completing rehabilitation training [33]. This study found that most of the respondents’ multidisciplinary early mobility teams had no clear division of labor. The reasons were as follows: ① Activities were not planned: There was no systematic activity plan, so the activities of patients were limited. Most of them stayed in bed or bedside for primary activities, and the requirements for human resources and professional members of the activity team were not high; ② Insufficient number of rehabilitation therapists: In order to save the time of patients’ lagging activities caused by waiting for the intervention of rehabilitation therapists, the work of rehabilitation therapists was partially replaced, and the professional boundaries of early mobility were gradually blurred. However, its implementation effect needs to be further investigated. ③Empiricism: The implementation process of early activities tended to be random, not based on standardized procedures, without scientific evaluation tools and implementation plans, unwilling to take risks and responsibilities, and seeking stability, which made high-level activities impossible or unfamiliar with the indications. The early activity of ICU patients is a continuous and teamwork process, and a clear division of labor guarantees safe and efficient implementation. It is impossible to carry out early activities in a standardized and continuous manner if the team members’ responsibilities, risks, and powers are unclear.

2) **Adopt practical training and practice modes**

A survey of 528 ICU medical staff found no significant difference in the cognition of ICU doctors, nurses, and respiratory therapists on the theory of early mobility in the ICU. However, only 55.9% of the medical staff participated in implementing early mobility [34]. This result reflects that they received the training of relevant theoretical knowledge and did not really “get moving.” Compared with the traditional training mode of “theory teaching + assessment,” the scientific team situational awareness education program was applied [35] or used multimedia and carried out workshops [36]. Learning and practicing in scenario simulation can better stimulate team members’ interest. In addition, multi-mode education that promotes information sharing among team members by holding meetings, lectures, or internal team discussions is a critical way to improve their knowledge reserve and practice. Practical training focuses on changing inefficient teaching methods rather than the task itself.

3) **Set up an appropriate incentive mechanism**

After the knowledge and concept of medical staff carrying out early activities for ICU patients have been improved, there is still a problem of insufficient action [37], partly due to the lack of a long-term incentive mechanism. Studies have shown that appropriate reward mechanisms (e.g., including the number of early activities performed in individual evaluation/performance evaluation indicators, giving appropriate team rewards, etc.) are as important as receiving relevant knowledge and skills training and reasonable allocation of human resources [35]. In addition, the lack of self-value in the team is also the internal cause of adverse actions [38]. At present, the role of essential members is weakened in implementing early mobilization in ICUs in China. Hence, the professionalism and effectiveness of early mobilization still need to be further verified [32]. Cultivating early activity specialized MDT teams with a clear division of labor can not only solve the problem of human resources shortages but also reflect the professional level of the team in the implementation process. Members can give professional opinions on the early activities and find their value while ensuring the implementation of the early activities.

(3) **Build a team collaboration model**

In the patient safety goals of the Chinese Hospital Association released in 2019, strengthening effective communication between medical staff was included as one of the top ten patient safety goals. In clinical practice, the team members’ unclear responsibilities and division of labor, the imperfect team operation mode, and the management mechanism led to a lack of communication among multidisciplinary members. Inevitable conflicts in rehabilitation, treatment, and nursing work processes become important factors affecting the effective implementation of early mobilization [17].

This study identifies critical gaps in ICU multidisciplinary collaboration—including unclear division of labor, poor communication, and ad hoc workflows—that directly hinder early mobilization efforts. These systemic flaws mirror catastrophic failures observed in surgical settings, where teamwork breakdowns result in preventable harm [39]. The parallels underscore a universal truth: patient safety across specialties depends on structured roles, verifiable accountability, and standardized protocols. To mitigate risks and optimize outcomes, ICU teams must adopt rigorous collaboration models, integrating surgical-style checklists, continuous training, and non-punitive reporting mechanisms. Only by institutionalizing these practices can we bridge the gap between theory and action, ensuring that early mobilization—like surgical safety—becomes an uncompromising standard of care.

### Limitations

This study focuses on the multidisciplinary teamwork approach in the early activities of mechanically ventilated patients in ICUs, and it does not apply to ICUs that have not yet developed multidisciplinary early activities for the time being. Secondly, the sampling method of this study was purposive sampling, a method that may lead to biased results that do not reflect their comprehensiveness. In order to control the bias as much as possible, ICUs in the central, eastern, and western regions were selected to make the results more convincing.

There may be potential bias in single-person interviews, and the subjectivity of the researcher may lead to unconscious bias. In this study, researchers were sensitive to their own biases during the interview and prepared recording equipment, pen and paper, etc., before the interview to record the interview content in detail. After the interviews, two researchers independently transcribed and coded the audio recordings. They then compared and discussed their results to enhance the objectivity and consistency of the data analysis. The interview transcripts were verified with the respondents, and the results were analyzed to ensure that the findings truly reflected their views and experiences.

## Conclusion

In this research, 22 members of the ICU multidisciplinary early mobility team were interviewed to learn about their feelings regarding cooperation and the challenges they faced when carrying out early mobility. It was found that there were problems such as a weak sense of teamwork, incomplete team building, and an imperfect team management mechanism. In the future, we can adjust the management concept (regarding resources, performance support, etc.), enhance the role of the multidisciplinary team, establish a team structure based on the cooperation requirements of medical staff, formulate continuous incentives or improvement measures, and optimize the team cooperation mode of ICU patients’ early activities so as to improve the efficiency of team cooperation and facilitate the implementation of early activities.

## Supporting information

S1 File Standards for Reporting Qualitative Research (SRQR). (DOCX)
